# Electric-field-controlled phase transition in a 2D molecular layer

**DOI:** 10.1038/s41598-017-07277-7

**Published:** 2017-08-04

**Authors:** Peter Matvija, Filip Rozbořil, Pavel Sobotík, Ivan Ošťádal, Barbara Pieczyrak, Leszek Jurczyszyn, Pavel Kocán

**Affiliations:** 1 Faculty of Mathematics and Physics, Charles University, Prague, 121 16 Czech Republic; 2 Instytut Fizyki Doswiadczalnej, Universytet Wroclawski, Wroclaw, 50-001 Poland

## Abstract

Self-assembly of organic molecules is a mechanism crucial for design of molecular nanodevices. We demonstrate unprecedented control over the self-assembly, which could allow switching and patterning at scales accessible by lithography techniques. We use the scanning tunneling microscope (STM) to induce a reversible 2D-gas-solid phase transition of copper phthalocyanine molecules on technologically important silicon surface functionalized by a metal monolayer. By means of ab-initio calculations we show that the charge transfer in the system results in a dipole moment carried by the molecules. The dipole moment interacts with a non-uniform electric field of the STM tip and the interaction changes the local density of molecules. To model the transition, we perform kinetic Monte Carlo simulations which reveal that the ordered molecular structures can form even without any attractive intermolecular interaction.

## Introduction

The process of molecular self-assembly, using organic molecules as building blocks of spontaneously grown molecular structures, became one of the leading topics important for development of molecular devices^1, 2^. In recent applications, such as organic field-effect transistors^3^, tunnel diodes^4^, solar cells^5^, light-emitting diodes^6^ etc., static ordered layers are utilized. A possibility to control molecular density—and thus ordering of whole molecular assemblies—by means of external stimuli would allow programmable molecular patterning^7^ at scales accessible by recent lithography techniques. Controlled switching has been well demonstrated on the level of isolated molecules^8–15^, but connecting the single-molecular switches to the rest of a functional device remains an obstacle^15, 16^. A small number of switchable ordered molecular structures reported up to date is limited to liquid-solid interfaces^17–22^ or small inorganic molecules^23^. In atomically clean environment under ultra-high vacuum (UHV), synchronized rotations of molecular rotors have been realized very recently^24^. However, to allow molecular patterning, switching of an assembly of mobile molecules mediated by surface diffusion would be necessary. So far, no such study in well defined UHV environment has been reported.

In our previous studies, we have shown that the Si(111)/Tl substrate is weakly interacting and suitable for growth of ordered ad-layers of metal atoms^25–27^. Compared to bare silicon surfaces, where adsorbed molecules are immobilized by strong chemical bonding to silion surface atoms^28–31^, the self-assembly of phthalocyanines on weakly interacting surfaces depends on a delicate balance between adsorbate-substrate and inter-molecular interactions, and on external parameters such as tempearture and surface coverage^32^. For growth of organic ordered layers composed of metalo-phthalocyanines (MPc), the weakly interacting metal surfaces are typically used as substrates^32^. A dominant factor governing the adsorption of different MPc molecules is a van der Waals interaction between the molecules and substrate, often resulting in a net static dipole moment perpendicular to the surface. Intermolecular interactions are weak attractive or even repulsive due to an interaction of the static dipoles, depending on the average inter-molecular distance^32–34^. The stable self-assembled layers are therefore often observed only at close-to-monolayer coverage, when the thermal motion of the molecules is limited by its neighbors^32, 35, 36^.

Here, we demonstrate the electric-field-controlled room-temperature switching of the copper phthalocyanine (CuPc) self-assembled arrays on the Si(111)/Tl - (1 × 1) surface, evidenced experimentally by the scanning tunneling microscopy (STM) and explained with help of ab-initio calculations. Stability of the ordered CuPc arrays during the scanning depends strongly on the polarity and amplitude of the applied tip-surface voltage. Negative sample bias stabilizes the ordered CuPc arrays, while application of positive sample biases causes the fast disintegration. By means of a strong electric field under the STM tip, we repeatedly change the orientation and position of all molecules within the domain. Measured probabilities of the domain changes demonstrate an existence of a voltage threshold above which the changes occur. Based on the results of ab-initio calculations and kinetic Monte Carlo (KMC) simulations, we provide a simple electrostatic model of the field-molecule interaction. The model explains the probabilistic nature of the process by disordering and the following random re-assembly of the molecular array at the measured threshold voltage. We expect that a similar electric-field control over surface assembly can be achieved for a wide range of systems containing a weakly interacting mobile adsorbate carrying sufficiently strong permanent dipoles.

## Results

### Switching of the self-assembled arrays

**Figure 1:**
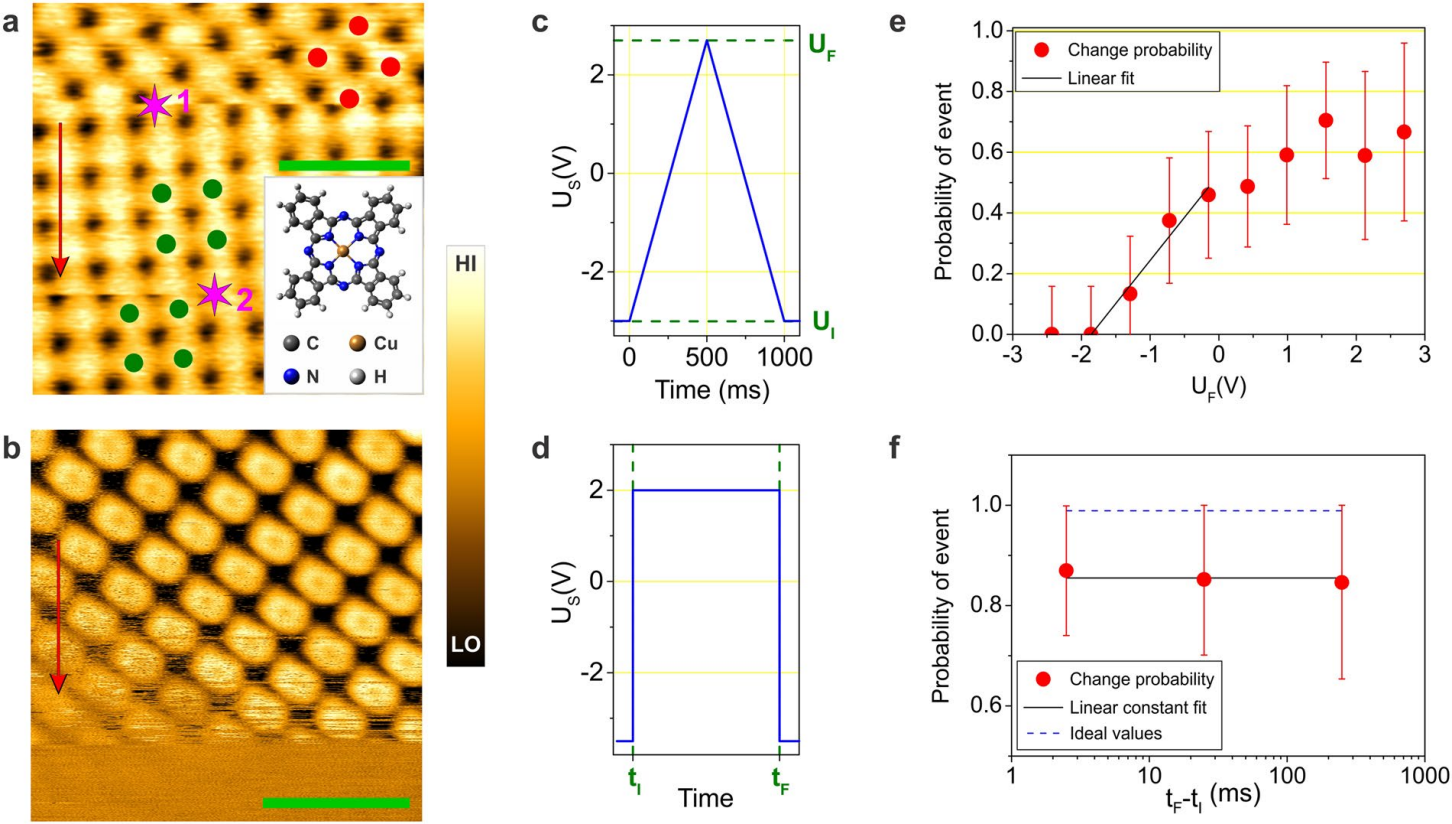
Field-induced switching of the CuPc self-assembled molecular array. (**a**) Change of a domain orientation (1) and position (2) as a response to voltage pulses. Purple stars mark positions above which the pulses were applied. The image was taken at the sample bias *U*_*s*_ = −2.3 V. The inset depicts the structural model of a CuPc molecule. Red and green dots mark positions of molecules in the molecular structures with different orientations. (**b**) STM image of the CuPc molecular array taken at the sample bias of −1.7 V, which is close to the threshold of imaging the stable arrays. Red arrows indicate the scanning direction. Green bars denote the length of 3 nm. (**c**,**e**) Shape of the triangular voltage pulse and corresponding dependence of the domain change probability on the peak voltage of the pulse *U*_*F*_. The values of probability were acquired from the 320 voltage pulses applied over the same domain. (**d**,**f**) Shape of the rectangular voltage pulse and corresponding dependence of the domain change probability on the pulse duration. The results were acquired from 100 voltage pulses applied over the same domain. Dashed line denotes ideal probability of random switching in a system of 45 domains (see Supplementary). The error bars represent a range of two standard deviations. Note that absolute values of the voltages and probabilities can vary for different STM tips and different surface coverages, but the character of the measured probability dependence remains the same.

**Figure 2:**
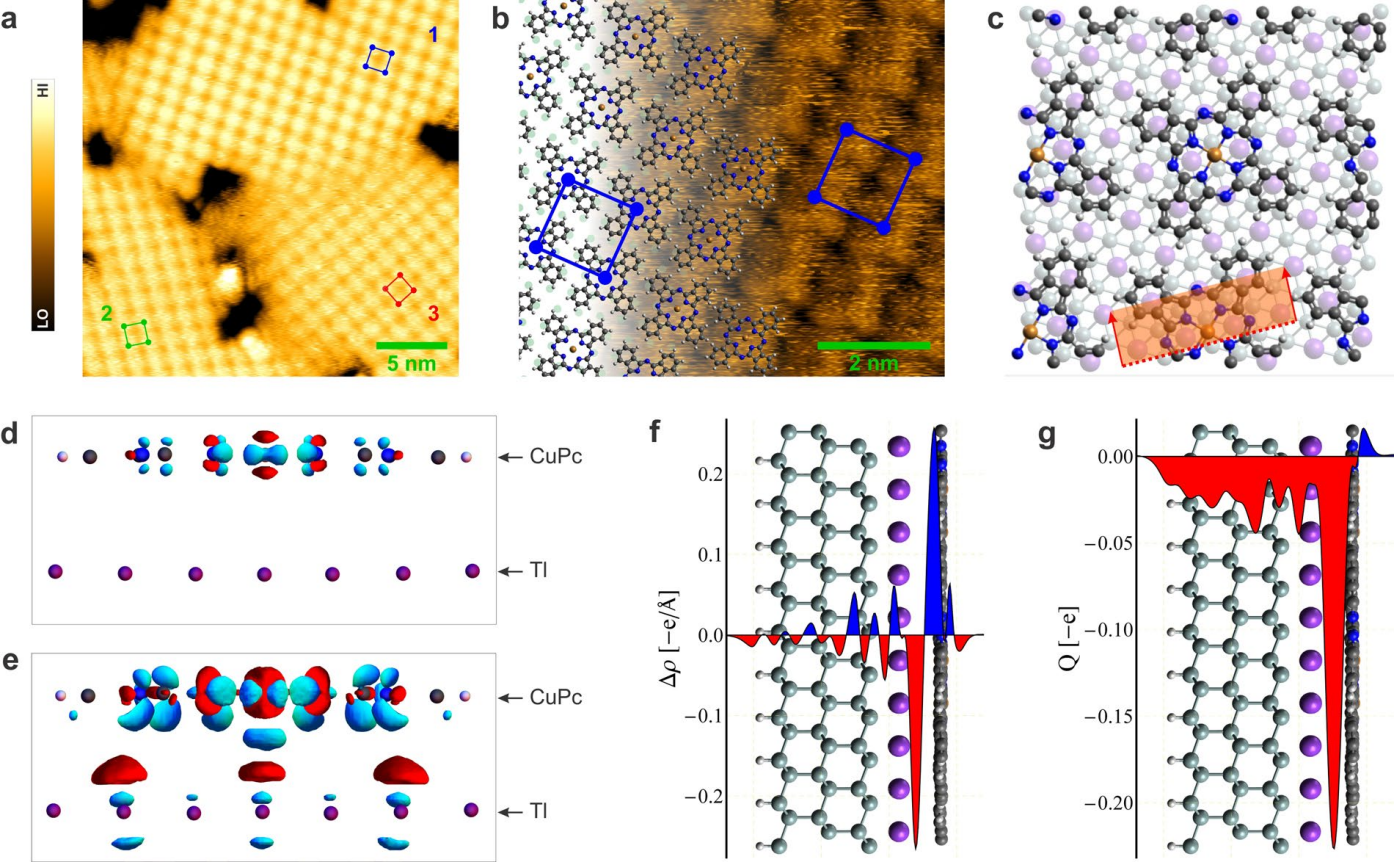
Self-assembly of CuPc molecules on the thallium-passivated Si(111) surface. (**a**) The Tl-passivated surface covered by almost 1 ML of CuPc molecules. Three domain orientations of the ordered CuPc array are shown. *U*_*s*_ = −3 V. (**b**) STM image of the CuPc array superimposed by the structural model. *U*_*s*_ = −2 V. Red, green and blue squares mark unit cells of the CuPc molecular arrays. (**c**) Top view of the relaxed structural model. Purple and gray spheres denote positions of Tl and Si atoms, respectively. (**d**,**e**) Isosurfaces of the constant difference of the charge density. The cross-section plane, the direction of the view and the volume imaged is marked in the panel c. The isovalues are set to 0.012 eÅ^−3^ (panel d) and 0.005 eÅ^−3^ (panel e). Red and blue colors represent electron density depletion and accumulation, respectively. (**f**,**g**) Side view of the relaxed structure superimposed by the plane-integrated charge transfer Δ*ρ* (panel f) and the cumulative charge transfer *Q* (panel g).

The CuPc molecule belongs to the family of planar macrocycles, phtalocyanines. These molecules tend to self-organize on nonreactive surfaces, as previously demonstrated using various substrates^13, 14, 32, 37–42^. STM images of approximately 0.8 monolayer (ML) of the CuPc molecules deposited on the Si(111)/Tl - (1 × 1) surface reveal the similar long-range ordering (Figs 1a,b and 2a,b). However, in addition to the self-assembly, we observe that the stability of the CuPc arrays at sub-monolayer coverage depends strongly on the scanning conditions. The ordered arrays are observable only at negative sample voltage *U*_*S*_ < −1.8 V, while scanning at sample voltage *U*_*S*_ > −1.8 V leads to noisy STM images without the presence of any ordered molecular structure. We note that the absolute value of the threshold can vary for different experimental conditions such as surface concentration of CuPc molecules, temperature etc. The presence of the threshold indicates that the electric field of the STM tip influences the molecular ordering. Figure 1b shows an STM image scanned at a sample voltage of −1.7 V, which is near the threshold of imaging the stable molecular arrays. During the scanning, the image of the initially stable CuPc structure is repeatedly perturbed by abrupt spikes until the ordered pattern completely disappears.

To reveal the mechanism of the tip influence we investigated the effect of tip-sample voltage pulses on the ordering of the molecules. During the image acquisition the scanning is stopped for a moment required by the pulse and then continues immediately. A result of such procedure is shown in Fig. 1a, where the purple stars mark positions where the pulses were applied. Abrupt changes in molecular layouts are visible immediately after the application of a pulse. More STM images of CuPc ordered arrays and their response to voltage pulses can be seen in Supplementary materials.

As we will show later in this work, the changes in the molecular layouts can be assigned to domain changes of the same CuPc ordering on the Tl-(1 × 1) surface. By repetitive applying different voltage pulses, we obtained statistics of the successful collective switching, which are present in Fig. 1e and f. We opted for two pulse shapes with various duration and amplitude. First, we applied triangular pulses (see Fig. 1c) with the initial voltage of −3 V (the stable ordered structure) to study dependence of the switching probability on the peak voltage *U*_*F*_. Though pulses with *U*_*F*_ in the range of (−3 V, −1.8 V) do not induce any change of the molecular arrays, from *U*_*F*_ ≈ −1.8 V the probability of the domain change increases approximately linearly with *U*_*F*_. The maximal switching probability (0.65 ± 0.10) is reached for sample biases ${U}_{F}\gtrsim 1.0\,V$. In the case of the rectangular pulse (see Fig. 1d), the sample voltage is changed from the scanning value of −3.5 V directly to + 2.0 V and is held constant for various time intervals. The average probability of a pulse-induced domain change saturates at the level of (0.9 ± 0.1). In the switching experiment, we were able to distinguish two types of the domain change. In the first case (Fig. 1a-1) the orientation of the molecular superlattice is changed, while in the second case (Fig. 1a-2) the orientation is preserved and the superlattice is shifted with respect to the original layout. For simplicity, the changes will be referred to as domain rotations and domain shifts, respectively. Taking into account the two categories, we analyzed the rectangular-pulse-induced changes and we determined that the average probability of the domain rotation is (0.6 ± 0.1) and the average probability of the domain shift is (0.3 ± 0.1). In the studied range from milliseconds to seconds, no dependence on the pulse duration was observed, implying that the time necessary for the switching is less than 1 ms.

Besides the external electric field, concentration of CuPc molecules and of surface defects have a significant impact on ordering of the molecules as well. The domain switching was observed only at near-monolayer surface coverage in the range of 0.6–0.95 ML. At saturated coverage, we observe stable molecular arrays at all scanning conditions. Here, CuPc molecules cover tightly the whole surface with exception of defects in the Tl- (1 × 1) layer and their surrounding (see Fig. 2a). At lower coverage, we observe only individual adsorption of CuPc molecules at step edges. Independently on STM conditions, no stable ordering of the molecules was observed on terraces up to ≈0.6 ML, indicating the presence of the 2D gas-like phase of highly mobile molecules on the surface. At all coverages, defects represent positions of the surface that are forbidden for CuPc molecules (see Supplementary materials for more STM images). Ordering of the molecules is substantially affected by defects especially if the local concentration of defects is high.

Existence of the voltage threshold of domain switching and absence of ordered molecular arrays at voltages above the threshold suggest that stability of the arrays depends on the external electric field. Changes of the tip-sample electric field during the pulse can explain switching of CuPc domains as follows. Molecular arrays are stabilized by the electric field at sample voltage ${U}_{s}\lesssim -1.8\,{\rm{V}}$. During the voltage pulse, at sample voltages corresponding to the saturated switching probability (${U}_{s}\gtrsim 1.0\,{\rm{V}}$), a whole domain of the CuPc array disintegrates faster than recordable by the STM. At the end of the pulse, when the voltage is restored to the original value, CuPc molecules reorganize randomly into one of the possible configurations (in total 90 different configurations exist; among them 45 configurations with 3 possible orientations are distinguishable in our experiment; see Supplementary for details). The expected probability of a domain change, rotation and shift in such a system would be $\frac{44}{45}$, $\frac{2}{3}$ and $\frac{14}{45}$, respectively. In the experiment, we obtained the average values of (0.9 ± 0.1), (0.6 ± 0.1) and (0.3 ± 0.1), respectively. All expected values fall within the standard deviation range of the measured values. Slightly lower mean experimental values are caused by surface defects which can locally “pin” or “block” some domains. Another reason is the limited room-temperature STM resolution, which could be insufficient to distinguish small domain shifts.

### Geometric and electronic structure of the ordered array

To clarify the details associated with the molecule-substrate and molecule-molecule interactions, the relativistic DFT calculations have been performed. In our models, we took into account the experimentally obtained Cu-Cu distance of the nearest molecules of ≈1.4 nm and the orientation of the molecules in the surface reconstruction shown in Fig. 2b. The remaining free parameter, the position of CuPc molecules with respect to the Tl-(1 × 1) surface, was determined by evaluating the adsorption energy of molecules in the most symmetric sites (on top, hollow and bridge) after relaxation of the testing configurations. The highest adsorption energy per molecule was obtained for central Cu atoms of CuPc molecules located above bridge positions of the Tl-(1 × 1) lattice (2.92 eV), followed by the hollow (2.83 eV) and on top sites (2.68 eV). In the relaxed bridge configuration (see Fig. 2c), molecules lie 0.33 nm above the Tl-(1 × 1) layer. Due to symmetries of the CuPc molecules and the bridge positions of the substrate, there are 90 equivalent CuPc configurations in total (see Supplementary for details).

On the Tl - (1 × 1) surface, the CuPc molecules are stabilized by van der Waals forces, which is also the case of phthalocyanine molecules on metal surfaces^32, 34^. Figure 2d and e show the charge transfer Δ*ρ* induced by an interaction of the CuPc molecule with the substrate. The transfer is calculated from the DFT calculations by subtracting charge densities of the isolated systems of the substrate and the adsorbate from that of the combined system. A dominant charge transfer is associated with Cu, N and C atoms in the central ring of the CuPc molecule (see Fig. 2d with isovalues ±0.012 eÅ^−3^). The transfer has a mirror symmetry plane, which is identical with the plane of the CuPc molecule and is parallel to the surface. Therefore, the transfer within the molecule contributes only to quadrupole or higher moments of the molecule.

Contributions to the dipole moment can be found mainly in the region between CuPc molecules and the Tl layer (see Fig. 2e with isovalues ±0.005 eÅ^−3^). The plane-integrated charge transfer Δ*ρ* (see Fig. 2f) shows that the charge is shifted from proximity of the surface towards molecules. In Fig. 2g, the cumulative charge transfer, obtained as $Q(z)={\int }_{-\infty }^{z}{\rm{\Delta }}\rho (z^{\prime} )dz^{\prime}$ is shown, indicating that ≈0.23 e is transferred in maximum, which results in a static dipole moment of ≈0.35 eÅ. Similar formation of a dipole pointing from the substrate to adsorbed molecules was calculated for other phthalocyanine-on-metal systems^32, 43^. In the following discussion, we neglect the complex charge transfer within the system and take into account only the dipole moments perpendicular to the surface plane.

### Mechanism of the phase switching

Under the studied conditions, the molecules are highly mobile on the surface. Two electrostatic forces acting on the molecules carrying dipoles can influence their dynamics: a dipole-dipole repulsive force and an interaction between the molecular dipole and the tip-sample electric field. The former can be neglected, because at the distance corresponding to the most close-packed configuration the interaction energy is ≈0.6 meV, which is much less than the energy of thermal fluctuations at room temperature *kT* ≈ 25 meV, where *k* is the Boltzmann constant. Presence of a biased STM tip in proximity of the surface induces a strong electric field (typically ≥10^7^Vcm^−1^) with maximum intensity *E* ≈ *U*/*d*_*TS*_ under the tip apex, where *d*_*TS*_ is the tip-sample spacing and *U* is the tip-sample voltage. Along the surface, the field is non-uniform and almost vanishes at a distance of the tip radius. The influence of the gradient field on mobile dipoles on surfaces has been previously reported^44^. In case of the positive (negative) tip bias and dipoles *μ* pointing perpendicularly to the surface, the field represents a potential well (hill) with depth (height) *μE* for each dipole. If the well is deeper than *kT*, an isolated molecule would be”trapped” by the tip. If a reservoir of molecules is available, their concentration would increase locally under the tip. Assuming the tip-sample separation of 1 nm, *μE* ≈ *kT* at a sample bias of ~−0.7 V. Because the high concentration results in ordering of the molecules, this value can be considered a threshold for assembly of the CuPc molecules into ordered arrays.

**Figure 3:**
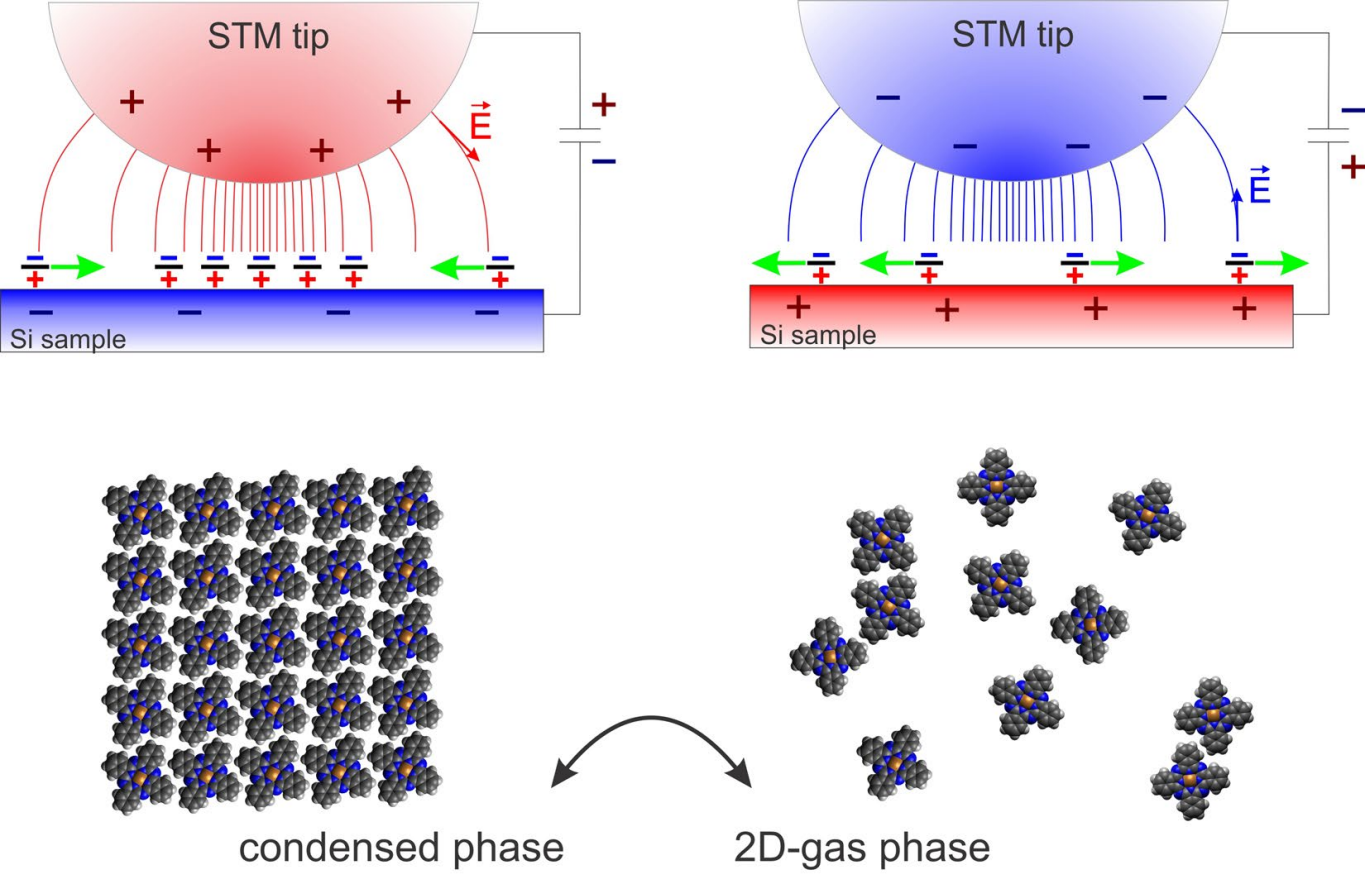
Graphic illustration of the field-controlled switching mechanism. Static dipoles carried by the CuPc molecules are represented schematically by the +/− signs on the surface. Green arrows indicate forces acting upon the dipoles in the electric field.

When scanning at the tip bias inducing sufficiently deep potential well, the locally increased concentration of molecules allows formation of a stable CuPc array (see Fig. 3). If we lower or reverse the bias (e.g. during a voltage pulse), the well becomes shallow or even reversed, which rapidly decreases the molecular concentration under the tip. At the end of the voltage pulse, when we sweep the voltage back to the scanning voltage, the potential well is restored and the molecules reorder randomly in the region under the tip to one of 90 possible configurations. This explanation is supported experimentally by Fig. 1e, where the voltage necessary for the CuPC assembly obtained from electrostatic relations and DFT calculations fits within the region of transition between zero and saturated switching probability, and by Fig. 1f, where the measured average switching probabilities correspond well to values expected in a system where domains switch into random domain layouts.

In order to further support the hypothesis that the existence of a potential well under the tip is sufficient for the self-assembly of molecules, we performed kinetic Monte Carlo simulations. In the simulations, the real shape of CuPc molecules is modeled by 15 lattice points of the substrate lattice and the interaction of molecules is limited to a steric repulsion which applies when the molecules overlap (see Supplementary). Apart from that, no inter-molecular interaction is included. Results of the simulations can be seen in Supplementary Fig. S6. On the intact surface the molecules at 0.75 ML coverage behave as a 2D gas (Fig. S6a and c). As a potential well is introduced to the surface, the local molecular coverage inside the well increases, inducing formation of the close-packed ordered molecular structure (Fig. S6b). The structure remains stable while the potential is held sufficiently low (Fig. S6d).

## Discussion

Our explanation neglects several factors. First, we consider a dipole moment only and omit higher moments of the molecule. The higher moments would affect a molecule-molecule interaction, which is though insignificant at RT, while the crucial interaction of the molecules with the tip field is driven by the dipole moment dominantly. Secondly, the strong field may affect molecule-substrate distance accompanied by a charge redistribution^45^. Such changes can be treated by the dipole approximation as well. If the dipole induced by the distance change was stronger than the static one, the tip-molecule interaction would be attractive at both bias polarities, in contrast to the experiment. Diffusion barriers can be modified by the field as well^45^, but we expect that at room temperature the diffusivity would be still very high, as supported by the field-independent 2D gaseous phase observed at coverage <0.6 ML. Thirdly, defects, such as step edges or point defects^46^, necessarily represent real boundary conditions, which may influence stability of ordered domains. In our experiments, we are able to find small areas isolated by defects where molecules are strongly confined and do not change their positions at any scanning conditions. Similarly, we are able to find areas with lower local concentration of molecules where we observe spontaneous switching even under otherwise stable conditions. Lastly, we note that experiments with different tips and coverage resulted in the qualitatively same results, however with different values of the threshold voltage in the range from −0.5 V to −2 V and different values of switching probabilities.

## Outlook

At sub-monolayer coverage and at room temperature, CuPc molecules behave like a 2D gas. We showed that, thanks to the permanent electric dipole between the molecules and the substrate, the local concentration of the molecules on the surface can be controlled by the nonuniform electric field. As ordering of the molecules is closely related to their local concentration, it is possible—at the scale of tens of nanometers—to switch between ordered and 2D-gas phases. An idea of patterning based on aggregation of molecules carrying dipoles by means of a nonuniform field has been introduced theoretically by Suo and Hong a decade ago^7^. Later, Jiang *et al*. reported possibility of tip-field-induced freezing of molecules on metal surface at 77 K, benefiting of increasing diffusion barriers rather than of dipole moments^45^. Our results show that it is possible to achieve the adaptive field-induced patterning at room temperature on technologically well mastered silicon substrates (see Supplementary Fig. S7).

In general, adsorption of molecules at surfaces is often accompanied by a charge transfer and formation of adsorption-induced permanent dipoles. We therefore expect that interaction of an external electric field with the dipoles can be utilized to control the surface assembly of a wide range of weakly-interacting adsorbates and substrates, similarly to the case presented in this study.

## Methods

### STM

Experiments were carried out in an ultra-high vacuum non-commercial STM apparatus with a base pressure below 5 × 10^−9^ Pa. Pressure during the experiments was sustained within the order of 10^−8^ Pa. The Si(111)/Tl - (1 × 1) surface was prepared by the thermal deposition of 1 ML of thallium (purity 99.999 %) on the Si(111) - (7 × 7) surface and annealing at 300 °C for 2 minutes^26^. The Si(111) - (7 × 7) surface was prepared on the Sb-doped Si monocrystal (resistivity 0.005–0.01 Ω.cm by flashing to 1200 °C. The samples were resistively heated by passing DC current. CuPc molecules were deposited on the passivated surface after cooling down the sample to the room temperature. Tunneling current was in the range 0.05–0.15 nA during the acquisition of all presented STM images.

### DFT

Our theoretical study is based on the density functional theory as it is implemented in the Vienna Ab-initio Simulation Package (VASP)^47–49^ and the use of plane wave basis set. In all presented calculations the description on the electron-ion interaction has been performed with the help of the projector-augmented wave (PAW) method while the exchange-correlation effects were included in the framework of generalized gradient approximation (GGA) in its Perdew-Burke-Ernzerhof (PBE) formulation^50^. The convergence of the energy of electronic states was performed using Davison-Block algorithm^51^ and the atomic structure has been relaxed using the conjugate gradient method. All calculations presented in the paper were performed with the use of nine k-points. The influence of this factor was tested in separate check calculations. The cutoff energy applied during calculations was 450 eV. The considered system was represented by a repeated slab model composed of CuPc molecule at the top, one Tl layer and six Si layers. The bottom Si layer was saturated by H atoms. In the total-energy calculations the molecule and 5 topmost layers of the substrate were relaxed until the forces present were less than 0.01 eV/Å. The slabs were separated by 15 Å of vacuum. The van der Waals interactions were included using the scheme of Grimme^52^.

### KMC

In the simulations, each molecule is represented by 15 lattice points which correspond to the real shape of CuPc molecules. The molecules can randomly either move to one of the 6 neighboring sites or rotate by 60°. Activation energy for both processes was calculated as the intersection point of neighboring harmonic potentials^53^. The energy of an adsorption configuration is increased by each lattice point in which two neighboring molecules overlap. Apart from that, no interaction was considered between non-overlapping molecules. The time-averaged maps of the local occupancy of lattice-points by molecules (see Supplementary Fig. S6) are obtained by averaging of the molecular positions over at least 1.5 × 10^6^ successive simulation steps per molecule.

## Electronic supplementary material

Supplementary information

## References

[1] Heath JR (2009). Molecular Electronics. Annu. Rev. Mater. Res..

[2] Claridge, S. A. *et al*. From the bottom up: dimensional control and characterization in molecular monolayers. *Chemical Society reviews* **42**, 2725–2745, http://pubs.rsc.org/en/content/articlehtml/2013/cs/c2cs35365b (2013). 10.1039/c2cs35365b PMC3596502 23258565

[3] Borshchev OV, Ponomarenko SA (2014). Self-assembled organic semiconductors for monolayer field-effect transistors. Polymer Science Series C.

[4] Celestin M, Krishnan S, Bhansali S, Stefanakos E, Goswami DY (2014). A review of self-assembled monolayers as potential terahertz frequency tunnel diodes. Nano Research.

[5] Ravi, S. K. & Tan, S. C. Progress and perspectives in exploiting photosynthetic biomolecules for solar energy harnessing. *Energy Environ. Sci*. **8**, 2551–2573 (2015).

[6] Sessolo M, Bolink HJ (2011). Hybrid organic-inorganic light-emitting diodes. Advanced Materials.

[7] Suo Z, Hong W (2004). Programmable motion and patterning of molecules on solid surfaces. Proceedings of the National Academy of Sciences of the United States of America.

[8] Pietzsch O (2007). Current-Induced Hydrogen Tautomerization and Conductance Switching of Naphthalocyanine Molecules. Science.

[9] Qiu XH, Nazin GV, Ho W (2004). Mechanisms of reversible conformational transitions in a single molecule. Physical Review Letters.

[10] Repp J, Meyer G, Olsson FE, Persson M (2004). Controlling the charge state of individual gold adatoms. Science.

[11] Haiss W (2003). Redox State Dependence of Single Molecule Conductivity. JACS.

[12] Yongfeng W, Kröger J, Berndt R, Hofer WA (2009). Pushing and pulling a Sn Ion through an adsorbed phthalocyanine molecule. Journal of the American Chemical Society.

[13] Stróżecka, A., Soriano, M., Pascual, J. I. & Palacios, J. J. Reversible change of the spin state in a manganese phthalocyanine by coordination of co molecule. *Phys. Rev. Lett*. **109**, 147202, http://link.aps.org/doi/10.1103/PhysRevLett.109.147202 (2012). 10.1103/PhysRevLett.109.147202 23083274

[14] Zhang JL (2014). Reversible switching of a single-dipole molecule imbedded in two-dimensional hydrogen-bonded binary molecular networks. Journal of Physical Chemistry C.

[15] Zhang JL (2015). Towards single molecule switches. Chem. Soc. Rev..

[16] Lörtscher, E. Wiring molecules into circuits. *Nature Nanotechnology* **8**, 381–384, http://www.nature.com/doifinder/10.1038/nnano.2013.105 (2013). 10.1038/nnano.2013.105 23736208

[17] Cui, K. *et al*. Potential-driven molecular tiling of a charged polycyclic aromatic compound. *Chemical Communications* **50**, 10376–10378, http://pubs.rsc.org/en/content/articlepdf/2014/cc/c4cc04189e (2014). 10.1039/c4cc04189e 25079562

[18] Cometto FP, Kern K, Lingenfelder M (2015). Local Conformational Switching of Supramolecular Networks at the Solid/Liquid Interface. ACS Nano.

[19] Lei SB (2008). Electric driven molecular switching of asymmetric tris(phthalocyaninato) lutetium triple-decker complex at the liquid/solid interface. Nano Letters.

[20] Zhang, X. M., Zeng, Q. D. & Wang, C. Reversible Phase Transformation at the Solid-Liquid Interface: STM Reveals. *Chemistry-an Asian Journal* **8**, 2330–2340 <Go to ISI>://WOS:000324748600008 (2013). 10.1002/asia.201300605 23825034

[21] Snegir SV, Yu P (2014). Switching at the Nanoscale: Light- and STM-Tip-Induced Switch of a Thiolated Diarylethene Self-Assembly on Au(111). Langmuir.

[22] Pace, G. *et al*. Cooperative light-induced molecular movements of highly ordered azobenzene self-assembled monolayers. *Proceedings of the National Academy of Sciences* **104**, 9937–9942, http://www.pnas.org/cgi/doi/10.1073/pnas.0703748104 (2007). 10.1073/pnas.0703748104 PMC1891213 17535889

[23] Wortmann B (2016). Reversible 2d phase transition driven by an electric field: Visualization and control on the atomic scale. Nano Letters.

[24] Zhang, Y. *et al*. Simultaneous and coordinated rotational switching of all molecular rotors in a network. *Nature Nanotechnology* **11**, 706–713, http://www.nature.com/doifinder/10.1038/nnano.2016.69 (2016). 10.1038/nnano.2016.69 27159740

[25] Matvija, P., Sobotík, P., Ošťádal, I. & Kocán, P. Diverse growth of Mn, In and Sn islands on thallium-passivated Si(111) surface. *Applied Surface Science* **331**, 339–345, http://www.sciencedirect.com/science/article/pii/S0169433215000914 (2015).

[26] Lee, S. S. *et al*. Structural and electronic properties of thallium overlayers on the Si(111) − 7 × 7 surface. *Phys. Rev. B* **66**, 233312, http://link.aps.org/doi/10.1103/PhysRevB.66.233312 (2002).

[27] Sakamoto, K. *et al*. Core-level photoemission study of thallium adsorbed on a Si(111) − (7 × 7) surface: Valence state of thallium and the charge state of surface si atoms. *Phys. Rev. B* **74**, 075335, http://link.aps.org/doi/10.1103/PhysRevB.74.075335 (2006).

[28] Papageorgiou N (2005). Physics of ultra-thin phthalocyanine films on semiconductors. Progress in Surface Science.

[29] Rochet F (1994). Copper phthalocyanine on Si(111) − 7 × 7 and Si(001) − 2 × 1: an XPS/AES and STM study. Surface Science.

[30] Krejčí, O. *et al*. Chemisorption of acetophenone on Si(111) − 7 × 7. polar aromatic molecule on electronically complex surface. *The Journal of Physical Chemistry C* **120**, 9200–9206, http://dx.doi.org/10.1021/acs.jpcc.6b00486 (2016).

[31] Tao F, Xu GQ (2004). Attachment chemistry of organic molecules on Si(111) − (7 × 7). Accounts of chemical research.

[32] Gottfried, J. M. Surface chemistry of porphyrins and phthalocyanines. *Surface Science Reports* **70**, 259–379, http://linkinghub.elsevier.com/retrieve/pii/S0167572915000114 (2015).

[33] Stadler, C., Hansen, S., Kröger, I., Kumpf, C. & Umbach, E. Tuning intermolecular interaction in long-range-ordered submonolayer organic films. *Nature Physics* **5**, 153–158, http://dx.doi.org/10.1038/nphys1176 (2009).

[34] Peisert, H., Uihlein, J., Petraki, F. & Chass, T. Charge transfer between transition metal phthalocyanines and metal substrates: The role of the transition metal. *Journal of Electron Spectroscopy and Related Phenomena* **204**, 49–60, http://dx.doi.org/10.1016/j.elspec.2015.01.005 (2015).

[35] Kröger, I. *et al*. Submonolayer growth of copper-phthalocyanine on Ag(111). *New Journal of Physics* **12** (2010).

[36] Stadtmüller B, Kröger I, Reinert F, Kumpf C (2011). Submonolayer growth of CuPc on noble metal surfaces. Physical Review B - Condensed Matter and Materials Physics.

[37] Menzli, S. *et al*. Adsorption study of copper phthalocyanine on Si(111) (√3 × √3) R30° Ag surface. *Applied Surface Science* **369**, 43–49, http://linkinghub.elsevier.com/retrieve/pii/S0169433216302161 (2016).

[38] Arbi I (2014). Molecular arrangement investigation of copper phthalocyanine grown on hydrogen passivated Si(111) surfaces. Applied Surface Science.

[39] Xiao K (2013). Surface-Induced Orientation Control of CuPc Molecules for the Epitaxial Growth of Highly Ordered Organic Crystals on Graphene. JACS.

[40] Floreano, L., Cossaro, A. & Gotter, R. Periodic arrays of Cu-phthalocyanine chains on Au (110). *J. Phys. Chem. C* **112**, 10794–10802, http://pubs.acs.org/doi/abs/10.1021/jp711140e (2008).

[41] Upward MD, Beton PH, Moriarty P (1999). Adsorption of cobalt phthalocyanine on Ag terminated Si(111). Surface Science.

[42] Wagner SR (2015). Growth of Metal Phthalocyanine on Deactivated Semiconducting Surfaces Steered by Selective Orbital Coupling. Physical Review Letters.

[43] Amin, B., Nazir, S. & Schwingenschlogl, U. Molecular distortion and charge transfer effects in ZnPc/Cu(111). *Sci Rep* **3**, 1705, http://www.ncbi.nlm.nih.gov/pubmed/23609223 (2013). 10.1038/srep03409 PMC3847700 24296477

[44] Whitman, L. J., Stroscio, J. A., Dragoset, R. A. & Celotta, R. J. Manipulation of Adsorbed Atoms and Creation of New Structures on Room-Temperature Surfaces with a Scanning Tunneling Microscope. *Science* **251**, 1206–1210, http://www.sciencemag.org/content/251/4998/1206.abstract (1991). 10.1126/science.251.4998.1206 17799280

[45] Jiang N (2010). Diffusivity control in molecule-on-metal systems using electric fields. Nano Letters.

[46] Kocán P, Sobotk P, Ošťádal I (2011). Metallic-like thallium overlayer on a si(111) surface. Phys. Rev. B.

[47] Kresse G, Hafner J (1993). Ab initio molecular dynamics for liquid metals. Phys. Rev. B.

[48] Kresse G, Hafner J (1994). Ab initio molecular-dynamics simulation of the liquid-metal21amorphous-semiconductor transition in germanium. Phys. Rev. B.

[49] Kresse G, Furthmüller J (1996). Efficient iterative schemes for ab initio total-energy calculations using a plane-wave basis set. Phys. Rev. B.

[50] Perdew JP, Burke K, Ernzerhof M (1996). Generalized gradient approximation made simple. Phys. Rev. Lett..

[51] Davison, E. R. *Methods in Computational Molecular Physics* (Plenum, New York, 1983).

[52] Grimme S (2006). Semiempirical GGA-type density functional constructed with a long-range dispersion correction. Journal of Computational Chemistry.

[53] Hood ES, Toby BH, Weinberg WH (1985). Precursor-mediated molecular chemisorption and thermal desorption: the interrelationships among energetics, kinetics, and adsorbate lattice structure. Phys. Rev. Lett..

