# Supplementary material for: Electric-field-controlled phase transition in a 2D molecular layer
Source: Sci Rep. 2017 Aug 4;7:7357. doi: 10.1038/s41598-017-07277-7 (PMC5544747; doi:10.1038/s41598-017-07277-7)
Supplement: Supplementary file 1 — Supplementary information [file 41598_2017_7277_MOESM1_ESM.pdf]

# Electric-field-controlled phase transition in a 2D molecular layer

(Supplementary information)

Peter Matvija[1*], Filip Rozbořil[1], Pavel Sobotík[1], Ivan Ošťádal[1], Barbara Pieczyrak[2], Leszek Jurczyszyn[2], Pavel Kocán[1]

[1] Faculty of Mathematics and Physics, Charles University, Prague, Czech Republic

[2] Institute of Experimental Physics, University of Wroclaw, Wroclaw, Poland

[*] corresponding author, email: matvija.peter@gmail.com

**Domain structure of the CuPc molecular array**

Based on the STM experiments and DFT calculations we were able to determine the structural model of the most stable dense configuration of CuPc molecules on the Tl-$1 \times 1$ surface (see Fig. 2 of the main article). To explain the switching experiment presented in Fig. 1 of the main article, it is important to understand the symmetry of the structure and its implications to number of possible configurations and number of configurations distinguishable in our STM experiments.

First of all, DFT calculations showed that the most stable position of the CuPc molecule on the Tl-$1 \times 1$ surface is a bridge site. To obtain the minimal energy, central Cu atoms of CuPc molecules have to sit directly above the bridge sites and their orientation must be as it is shown in Fig. 2c in the main article. In order to create the molecular array with the minimal energy, all molecules of the same domain have to have the same orientation. There are 3 possible orientations of CuPc molecules corresponding to 3 different bridge positions within a $1 \times 1$ Tl cell (see Fig. S1a and Fig. S1c-e). Since CuPc molecules and bridge positions have common mirror symmetry (shown in Fig. S1), there are two different ways the molecules can be arranged on the substrate at each of the three orientations. The two different arrangements are marked by two different colors at each panel c-e. In the figure, only positions of central Cu atoms of CuPc molecules are marked and only one central molecule is fully depicted. Orientation of the rest of the molecules is the same as the orientation of the central molecule. Super-cells of the molecular arrays are marked by red, green and blue squares. If we count only configurations where central CuPc molecules sit above one given unit cell of the Tl-$1 \times 1$ lattice, we obtain $3 \times 2 = 6$ possible configurations. A super-cell of the CuPc molecular array, however, contains 15 unit cells of the Tl-$1 \times 1$ lattice (see Fig. S1b). Therefore the total number of configurations of the CuPc molecular array equals to $3 \times 2 \times 15 = 90$.

In our STM experiments, a dominant feature of CuPc molecule imaging at room temperature and $U_s \sim -3$ V is its central C-N macrocycle (see Fig. S5c). As a consequence, we are often not able to determine the orientation of individual molecules. Domains with the same orientation of the molecular superlattice, but the different orientation of individual molecules are percieved as

identical (see the different domains marked by the same color in Fig. S1c-S1e). The identity reduces the total number of 90 domains to 45 distinguishable domains and the total number of domain orientations to 3 distinguishable orientations.

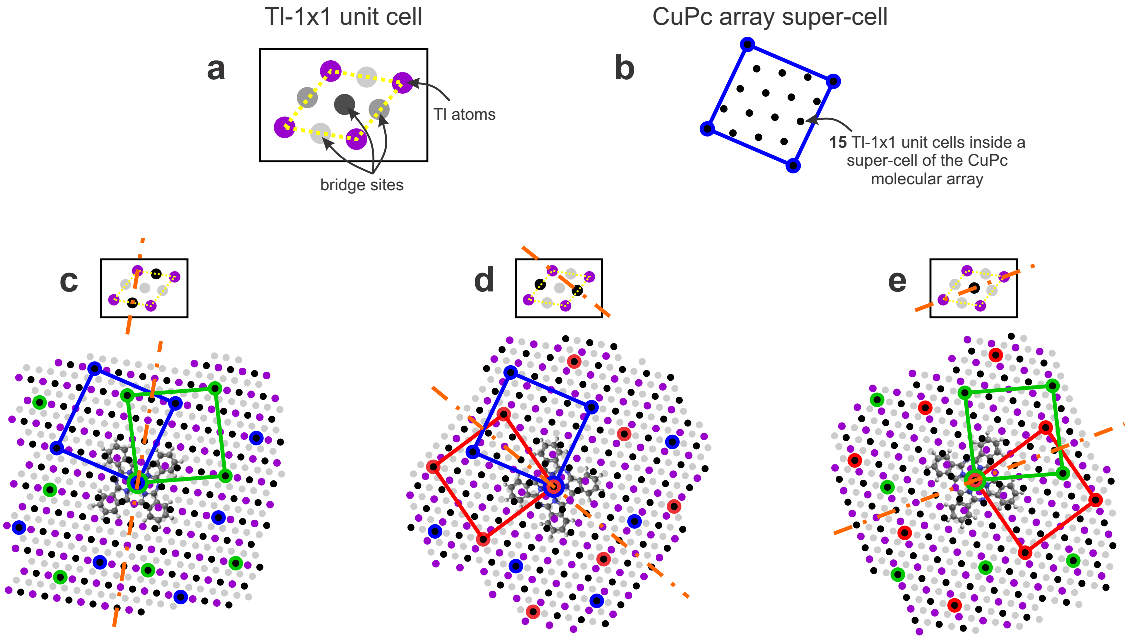


Figure S1: **Possible configurations of the CuPc molecular array on the Tl-$1 \times 1$ surface. a**, Unit cell of the Tl-$1 \times 1$ surface reconstruction. Positions of Tl atoms are marked by purple dots. Gray dots represent 3 bridge sites with different orientation in the unit cell. **b**, Example of a super-cell of the CuPc molecular array on the Tl-$1 \times 1$ surface. Black dots mark positions of Tl-$1 \times 1$ unit cells inside the super-cell of the CuPC array. **c-e**, Schematic drawing of possible configurations of CuPc molecular array on the Tl-$1 \times 1$ surface. Each panel corresponds to one possible orientation of CuPc molecules on the surface. Bridge sites that can be occupied by a central Cu of a CuPc molecule in a given orientation are marked by black dots. Bridge sites corresponding to a different orientation of the molecules are marked by gray dots. There are two possible molecular arrangements in case of each orientation of the molecules (marked by two different colors on each panel). Positions of central Cu atoms of CuPc molecules are marked by red, green and blue dots. Unit cells of the molecular arrays are marked by squares of the same color. The same colors in different panels indicate the same orientations of the whole molecular arrays.

**Domain switching viewed by STM**

Figures S2 - S4 display dynamic behavior of CuPc domains during the STM scanning. All images were acquired at $U_s < -2$ V, which is above the switching threshold (i.e. the voltage is sufficiently high to stabilize CuPc domains). After application of voltage pulses which penetrate below the threshold, sudden domain changes are observed. Fig. S2 illustrates the domain changes on a large area. The imaged area is initially covered by a single domain of the CuPc molecules. The orientation of the domain is marked by blue lines. After application of a voltage pulse (top purple star), the right part of the domain is shifted (red lines), while the left part remains without a change. Domain boundary emerges between the two domains. The second pulse induces rotation of both domains.

More detailed images of the domain switching are presented in Fig. S3. Note that a big fraction of the pulses, especially in Fig. S3f, does not induce any domain change. We observe this effect on areas with high density of defects which represent boundary condition for the ordering of molecules and can pin or prevent certain domains.

The influence of defects on CuPc ordering is even more pronounced in Fig. S4. Here high density of defects induces stabilization of domains on certain areas (marked by 1a), while other areas remain covered by unstable and spontaneously switching domains (marked by 2). Areas where defects prevent formation of stable domains and areas where defects hinder aggregation of molecules from surrounding surface are covered by a 2D molecular gas (marked by 3). At the interface of the stable domains and the gas, gradual decrease of the apparent molecular brightness can be seen. The lower brightness of the molecules can be explained as lower probability to find a molecule at a given position. The lower probability arises as a result of the dynamic equilibrium between the gas and condensed phase and it is the clear evidence of presence of the 2D gas phase on the surface.

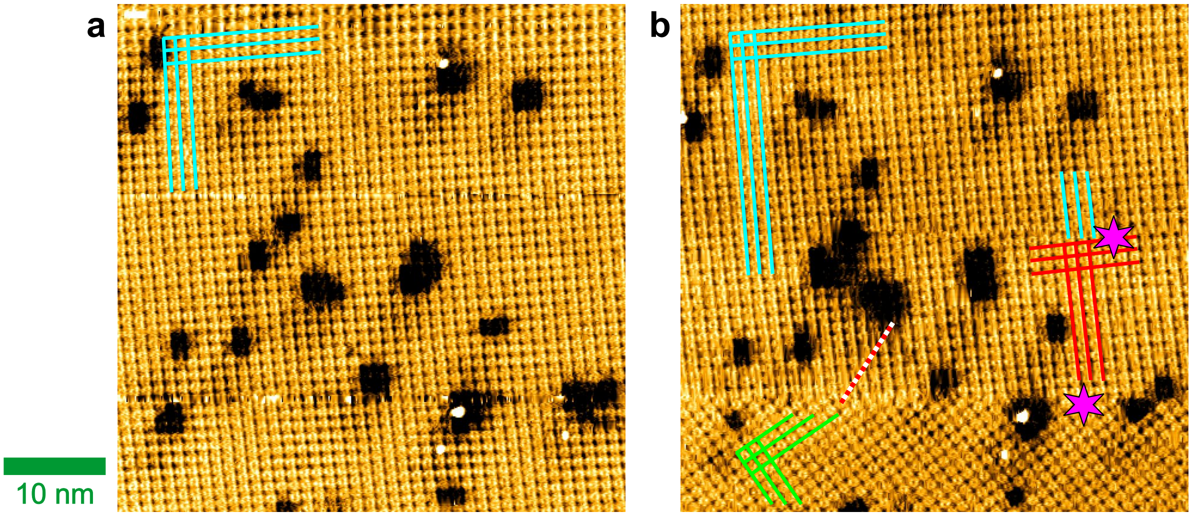


Figure S2: **Domain switching on a large area.** Two successive STM images of the same area are displayed. Differently colored solid lines mark orientations of different domains. Red-white line marks a domain boundary. Purple stars on the panel **b** marks positions where triangular voltage pulses ($U_f = 1$ V) were executed. $U_s = -3$ V. Note that (i) images were not corrected for a thermal drift and piezo-creep and that (ii) positions of defects remain unchanged after the pulses. Scanning direction is from the top to the bottom.

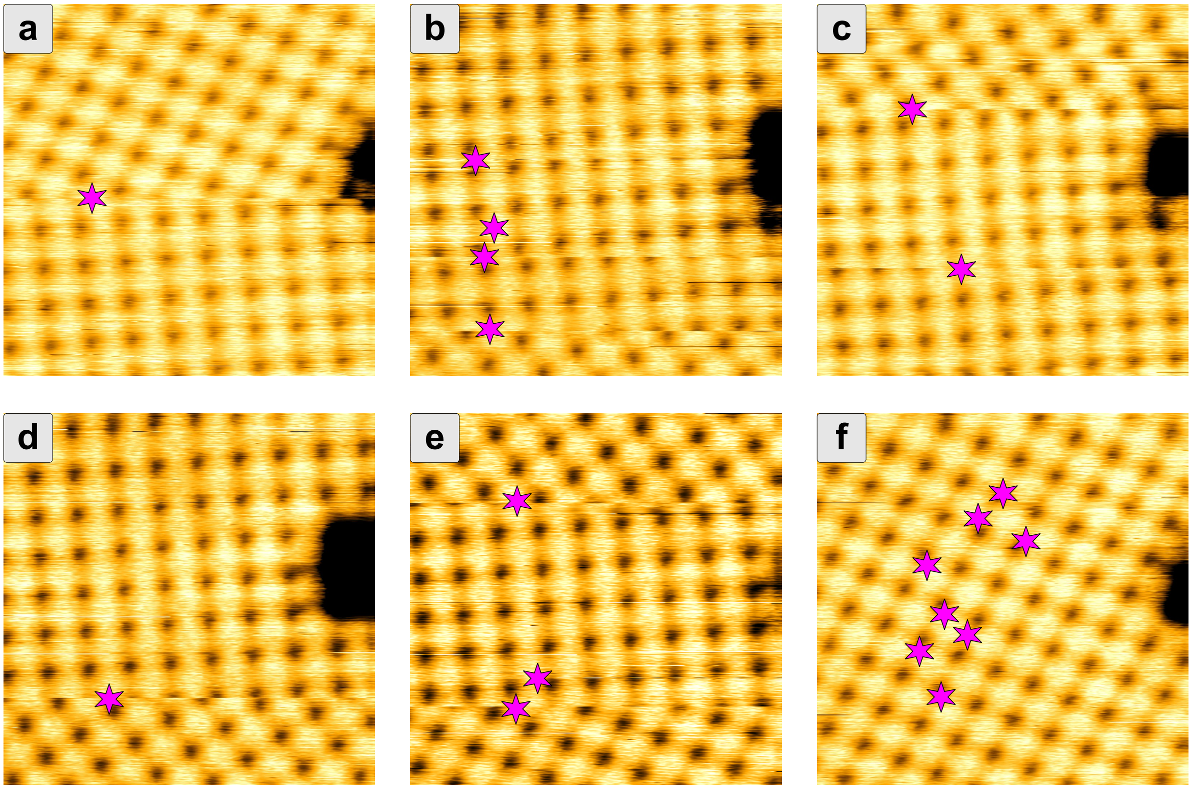


Figure S3: **Multiple domain switching.** Six consecutive STM images, $U_s = -2.3$ V, showing abrupt domain changes after voltage pulses. 19 triangular voltage pulses from -2.3 V to 0 V were executed. 9 domain changes were identified. Positions of the voltage pulses are marked by purple stars. Note that slight deviations in the position of the dark defect are caused by inaccurate manual compensation of the thermal drift. Size of the imaged area is 10 nm$\times$10 nm. Scanning direction is from the top to the bottom.

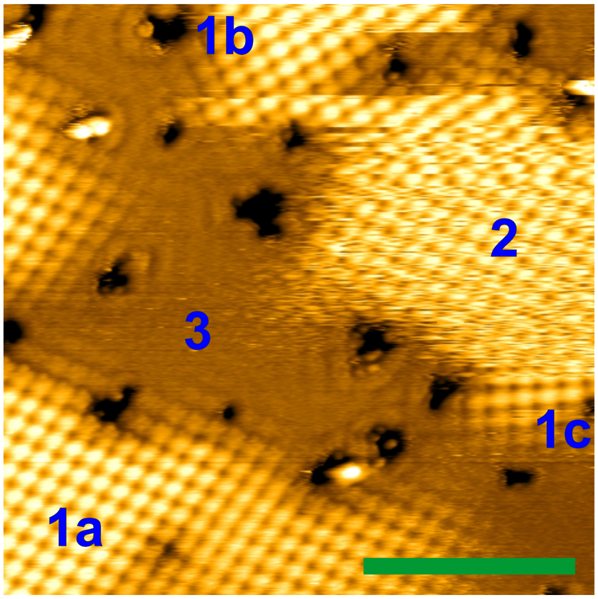


Figure S4: **Coexistence of the 2D gas and condensed phase on the surface. 1a**, **1b** and **1c** mark three possible orientations of stable CuPc domains. **2** marks an unstable CuPc domain which is spontaneously switching during the STM measurement. Area between the domains is filled by a 2D gas (marked by **3**). Surface coverage $\approx 0.7$ ML. $U_s = -2.5$ V. Green bar denotes 10 nm.

**DFT calculations**

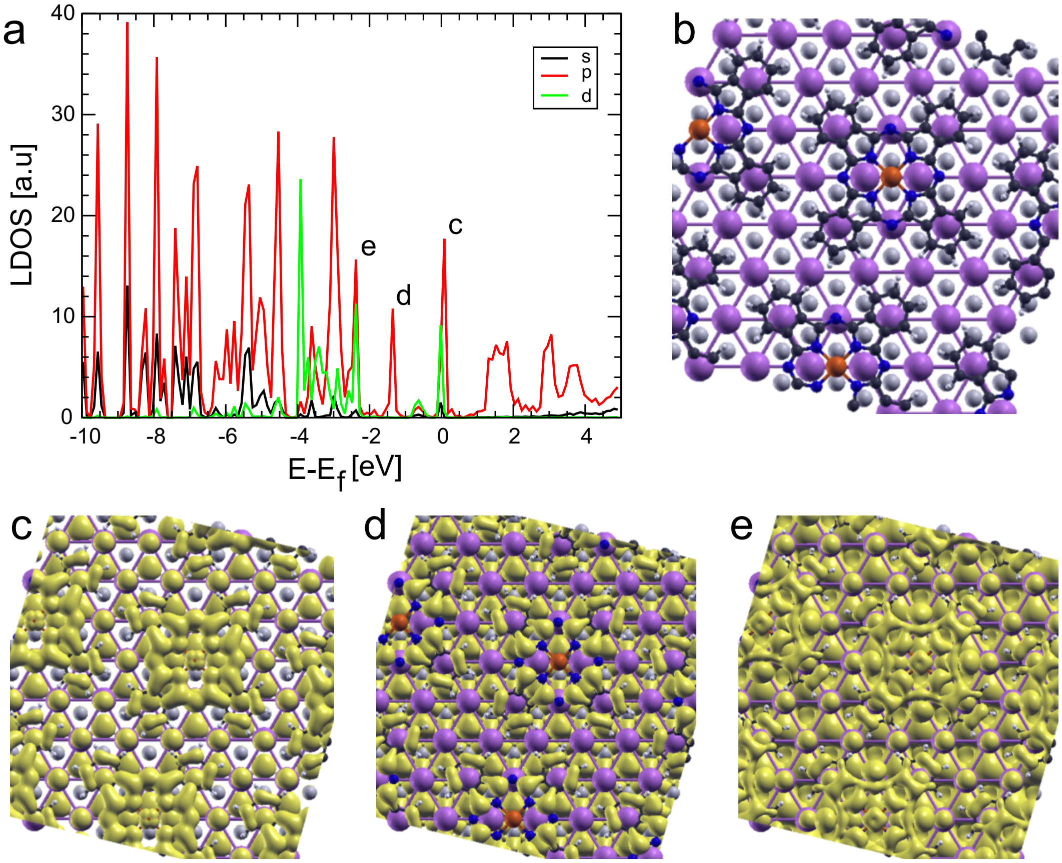


Figure S5: **a**, Calculated local density of states (LDOS) of the CuPc molecule deposited on the Si(111)/Tl-(1x1) surface projected on the s (black line), p (red line) and d (green line) orbitals of the molecule. **b**, Top view of the corresponding system. **c**, **d**, **e**, Partial charge distributions of the LDOS features shown in the panel **a**; isosurface values are 0.0035, 0.0035 and 0.002 eÅ$^{-3}$, respectively.

**KMC simulations**

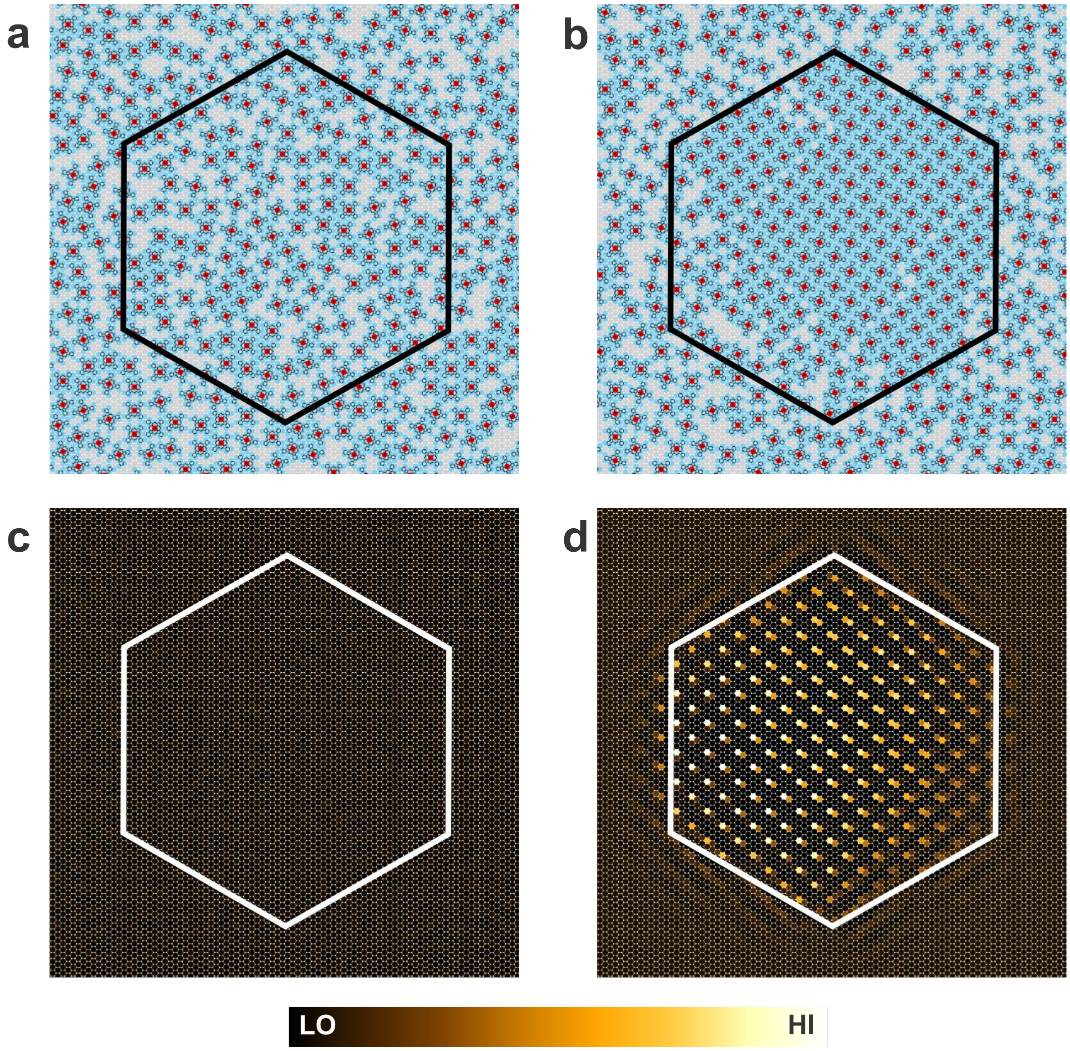


Figure S6: KMC simulations of the molecular condensation in the STM-tip-induced potential well on the surface with 0.75 ML molecular coverage at a temperature of 300 K. The figure displays snapshots of KMC simulations without (**a**) and with (**b**) the 0.075 eV potential well and the corresponding maps of the time-averaged lattice-points occupancy (**c** and **d**, respectively). The hexagonal potential well is outlined by black and white lines. The well has a rectangular cross-section, i.e. the adsorption energy per one lattice point is increased for every lattice point inside the well by the constant value of 0.075 eV. Close-packed ordering of molecules in the potential well can be clearly seen even without any attractive interaction between CuPc molecules.

**Stable switchable molecular arrays**

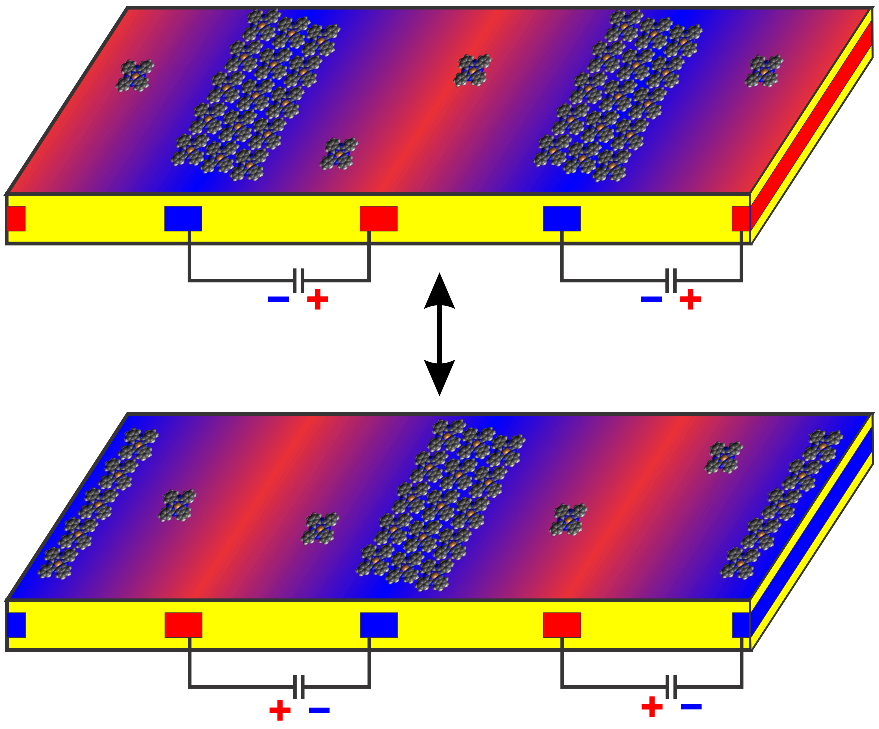


Figure S7: The proposition of a stable switchable molecular array consisting of Tl-covered undoped silicon layer (yellow), conducting buried electrodes (red and blue) and mobile CuPc 2D gas on top of the surface. Red and blue colors of the surface represent areas of potential hills and wells, respectively.
